# Contrasting vulnerability of monospecific and species‐diverse forests to wind and bark beetle disturbance: The role of management

**DOI:** 10.1002/ece3.6854

**Published:** 2020-10-16

**Authors:** Laura Dobor, Tomáš Hlásny, Soňa Zimová

**Affiliations:** ^1^ Faculty of Forestry and Wood Sciences Czech University of Life Sciences Prague Prague Czech Republic

**Keywords:** bark beetles, Central Europe, climate change, disturbance management, salvage logging, simulation model, tree species diversity

## Abstract

1. Wind and bark beetle disturbances have increased in recent decades, affecting Europe's coniferous forests with particular severity. Management fostering forest diversity and resilience is deemed to effectively mitigate disturbance impacts, yet its efficiency and interaction with other disturbance management measures remain unclear.
2. We focused on Central Europe, which has become one of the hotspots of recent disturbance changes. We used the iLand ecosystem model to understand the interplay between species composition of the forest, forest disturbance dynamics affected by climate change, and disturbance management. The tested measures included (a) active transformation of tree species composition toward site‐matching species; (b) intensive removal of windfelled trees, which can support the buildup of bark beetle populations; and (c) reduction of mature and vulnerable trees on the landscape via modified harvesting regimes.
3. We found that management systems aiming to sustain the dominance of Norway spruce in the forest are failing under climate change, and none of the measures applied could mitigate the disturbance impacts. Conversely, management systems fostering forest diversity substantially reduced the level of disturbance. Significant disturbance reduction has been achieved even without salvaging and rotation length reduction, which is beneficial for ecosystem recovery, carbon, and biodiversity.
4. *Synthesis and applications:* We conclude that climate change amplifies the contrast in vulnerability of monospecific and species‐diverse forests to wind and bark beetle disturbance. Whereas forests dominated by Norway spruce are not likely to be sustained in Central Europe under climate change, different management strategies can be applied in species‐diverse forests to reach the desired control over the disturbance dynamic. Our findings justify some unrealistic expectations about the options to control disturbance dynamics under climate change and highlight the importance of management that fosters forest diversity.

## Graphical abstract

We contrasted disturbance patterns of a Central European mountain forest that undergoes an intense transformation of species composition against a forest, where management maintained the dominance of vulnerable Norway spruce. Our findings justify some unrealistic expectations about the options to control disturbance dynamics under climate change and highlight the importance of management that fosters forest diversity.

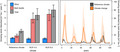

## 1 INTRODUCTION

Forest disturbances are an integral part of forest dynamics, contributing to ecosystem functioning, creating heterogenous landscapes, and fostering biodiversity (Beudert et al., 2015; Turner et al., 2004). In production forests, however, disturbances place social management objectives at risk and compromise the provision of valued ecosystem services (Lindroth et al., 2009; Seidl & Blennow, 2012). Research suggests that all types of ecosystem services are negatively affected (Thom & Seidl, 2016), and these impacts will continue to increase in the future (Seidl, Schelhaas, Rammer, & Verkerk, 2014). Efforts to prevent or mitigate disturbance impacts have therefore become an integral part of forest management in Europe. The applied measures include, for example, improvement of tree vigor and morphology, modification of stand structure and composition, or reduction of fuel loads and breeding substrate for insects (Berryman, 1988; Gardiner & Quine, 2000; Jactel et al., 2009; Wermelinger, 2004). Research has also highlighted some controversies related to active disturbance management. These particularly include an effort to replace complex ecosystem regulation dynamics by oversimplified technological processes, which often erode ecosystem resilience (Cox, 2016) and produce collateral effects interfering with local management objectives (Leverkus, Lindenmayer, Thorn, & Gustafsson, 2018). For example, long‐term outbreak prevention via salvage logging can increase forest vulnerability to future disturbances via creation of vulnerable complexes of mature stands with high growing stock (Dobor et al., 2020). Intensive disturbance management can also affect the quality of ecosystem services and modify natural ecological interactions in the forests (Leverkus, Lindenmayer, et al., 2018).

Forest disturbance management has received increased attention in response to the recently intensified disturbance regimes and the increased rate of social and ecological impacts (Müller, 2011; Senf et al., 2018). Moreover, model projections indicated that disturbance intensification will continue to increase in the future, which highlights the need to revise current management strategies (e.g., Dobor et al., 2019, 2020; Hlásny et al., 2019; Honkaniemi et al., 2020; Kausrud et al., 2012; Seidl et al., 2018). This requires a comprehensive understanding of the interactions between vegetation and disturbance dynamics affected by climate change, and management, which strives to interact with this overly complex and potentially unstable system. Quantifying the outcomes of disturbance management in ecosystems affected by climate change is therefore beyond our current understanding, which was mostly developed under past and more stable conditions.

Among different disturbance agents, bark beetles (Coleoptera: Curculionidae, Scolytinae) have shown remarkable climatic sensitivity (Cudmore et al., 2010; Seidl & Rammer, 2017). Recent intensification of bark beetle disturbance in Europe has been greater than that of any other disturbance type, including wind and wildfires (Seidl, Schelhaas, et al., 2014). While total canopy mortality has doubled in Europe over the last three decades (Senf et al., 2018), impact from bark beetles has increased by 600% (Seidl, Schelhaas, et al., 2014). These outbreaks highlight the prominent role of climate change as the driver of bark beetle disturbance (Bentz et al., 2019; Jönsson et al., 2009). In Europe's *Picea abies—Ips typographus* system, climate change increases the number of bark beetle generations, reduces winter mortality, and compromises fitness of host trees (Huang et al., 2019). Climate change also synchronizes the outbreaks over areas large several hundreds of kilometers via the large‐scale impacts of regional climate extremes (Senf & Seidl, 2018). Outbreaks of *I. typographus* amplified by climate change thus represent one of the major threats to forestry economies and the environment in Europe (Grégoire et al., 2015; Komonen et al., 2011).

In Europe's production forests, management has traditionally strived to control bark beetle populations to prevent or mitigate their impacts (Berryman, 1988; Wermelinger, 2004). These measures either aim to directly control populations of bark beetles or to modify forest structure and composition to create environment that is less conducive to the outbreaks (Wermelinger, 2004). Direct control mainly endeavors to reduce the amount of breeding substrate for beetles by removing trees affected by wind, snow, and rime; remove infested trees from the forest prior to beetles’ emergence; and reduce beetle populations using insecticides or various trapping devices (Faccoli & Stergulc, 2008; Stadelmann et al., 2013). Conversely, indirect control includes silviculture practices, which, for example, aim to reduce tree competition for resources using thinning, reduce the concentration of host trees via change in tree species composition, or modify harvesting regimes to reduce the share of mature and vulnerable trees (Björkman et al., 2015; Jactel et al., 2009; Zimová et al., 2020). Indirect control can also aim to modify the forest configuration on the landscape to reduce the connectedness of local bark beetle populations and complexes of host trees (Honkaniemi et al., 2020; Seidl et al., 2018).

Efficiency of outbreak management measures in reducing the level of tree mortality is generally not sufficiently understood to inform management decisions (Hlásny et al., 2019; Kausrud et al., 2012). Rare examples of quantitative assessments include the studies of Faccoli and Stergulc (2008) for pheromone traps, and Stadelmann et al. (2013) and Dobor et al. (2019, 2020) for salvage logging. This lack of quantitative understanding becomes critical if the outbreaks are amplified by climate change and management resources are becoming increasingly limited. Still, the consensus exists that species‐diverse forest with complex structures show increased resistance to herbivores (Guyot et al., 2016) and have higher survival rates (Griess et al., 2012; Neuner et al., 2015). Adaptive change in tree species composition can dilute the host trees in the forest, increase semiochemical diversity, and strengthen resilience mechanisms (Seidl, 2014; Zhang & Schlyter, 2003). Managing forests for diversity is thus recognized as a prominent strategy to mitigate bark beetle disturbance. Because the effect of silviculture management can be rather delayed, it can be applied concurrently with other measures such as salvage removal of windfelled trees, beetle trapping, or premature harvesting of vulnerable stands. Interactions of these effects may, however, generate hardly predictable nonadditive outcomes, which can be further modulated by climate change (Dobor et al., 2020).

Here, we investigate how management of functionally linked wind and bark beetle disturbances performs in differently managed forests, and how this performance can be affected by climate change. In particular, we investigated how adaptive change in tree species composition affects the vulnerability of forests dominated by *P. abies* to natural disturbances and how the transformation of species composition interacts with other disturbance management measures. We focused on Central Europe, which has become one of the hotspots of recent disturbance change, and where the revision of current disturbance management strategies is urgently required. Because this analysis requires considering landscape‐scale climate‐sensitive disturbance dynamics, disturbance interactions, and dynamic feedback from vegetation, we addressed these questions using the forest landscape and disturbance model iLand (Seidl et al., 2012).

## 2 DATA AND METHODS

### 2.1 Study region

**FIGURE 1:**
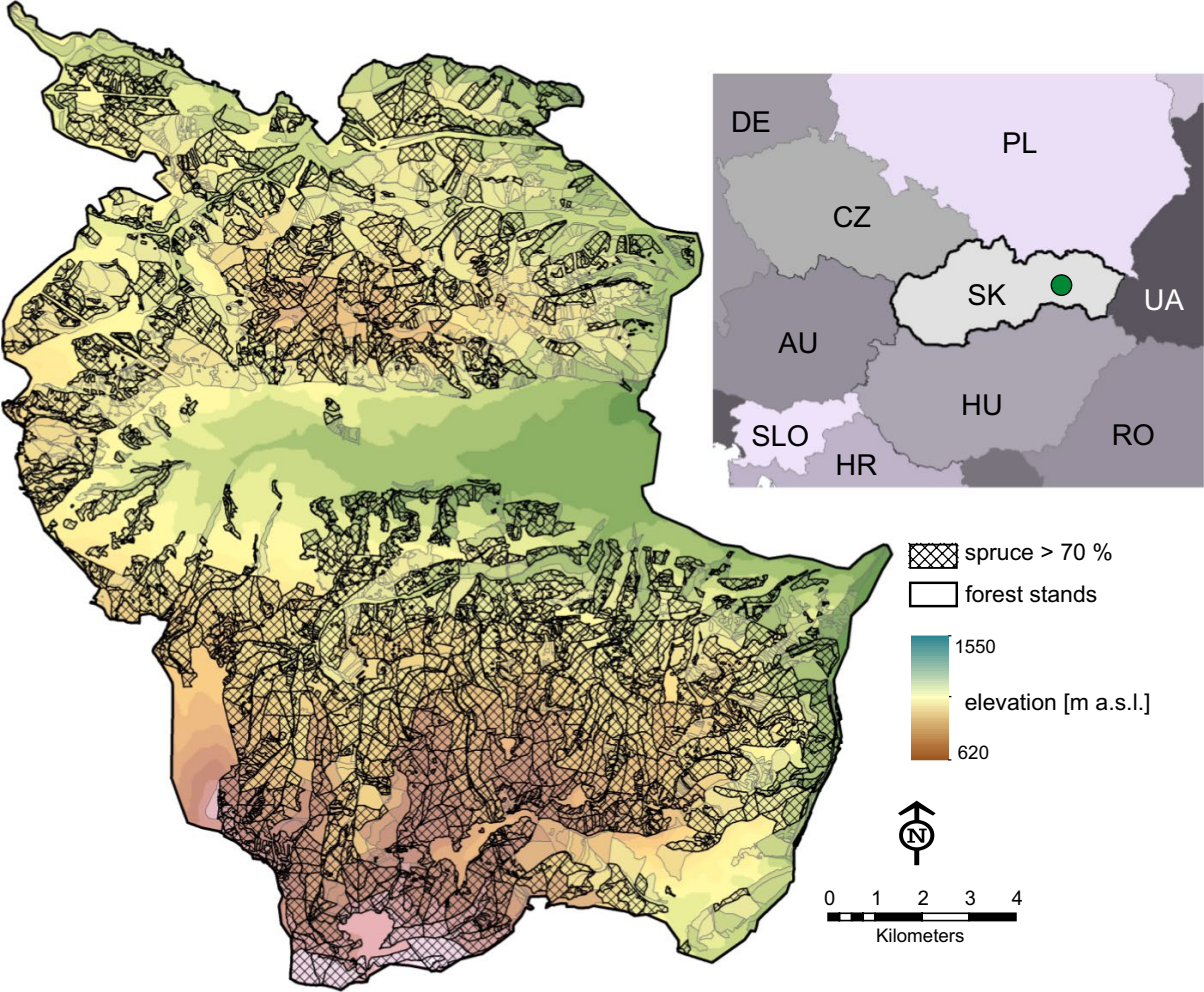
The study area: the Goat Backs Mt. landscape. Forest distribution and stands with the dominance of Norway spruce are indicated. Elevation is displayed in the background. The insert shows the location of the study landscape in Central Europe (green circle)

The study area is in the Western Carpathians (the Low Tatras mountain range) in Slovakia (Lon 20.088–20.275, Lat 48.920–49.061), covering an area of 16,050 ha (Figure 1). The landscape has 70% forest cover and elevation range of 620 to 1550 m a. s. l. The forests are chiefly managed for softwood timber production, though recreation, game management, and nature conservation also occur.

Intense elevation and climate gradients, and the temperate continental climate (Kottek et al., 2006) resulted in the presence of multiple zonal forest communities in the natural species composition, with a dominance of broadleaved species (Rizman et al., 2005). Due to the intense production‐oriented management applied over the last 200 years, however, the forests are currently dominated by Norway spruce (*Picea abies*), which is found (often in monocultures) across the majority of site types on the landscape (Figure 1). Other important tree species are European larch (*Larix decidua* Mill.), Scots pine (*Pinus sylvestris* L.), Silver fir (*Abies alba* Mill.), and European beech (*Fagus sylvatica* L.).

The current silvicultural system is an even‐aged management regime with a rotation length of approximately 100 years. The primary approach to tree regeneration in stands with fir and/or beech admixtures is a uniform shelterwood cut, that is, progressive cutting that leads to the establishment of a new cohort of trees under the canopy of the retained mature trees. The shelterwood system contains 3 to 4 regeneration cuts applied over a period of approximately 30 years, followed by a final cut. In spruce monocultures, a small‐scale clearcutting system is applied (cut‐block size < 3 ha).

Recent years have been characterized by high natural disturbance activity, followed by high levels of salvage and sanitation felling. The natural disturbance regime consists of bark beetle (mainly *Ips typographus* L.) and wind disturbance, which has considerably intensified over the last 20 years.

### 2.2 Simulation model

We investigated the interactions between the climate, management, disturbances, and vegetation dynamics using the process‐based model iLand (Seidl et al., 2012) (http://iland.boku.ac.at). iLand is an ecosystem model that simulates forest landscape dynamics, including growth and regeneration, disturbance dynamics, and management in a spatially explicit manner. The main entity in the model is a tree, for which the demographic processes are simulated. Processes at the stand and landscape scale constrain the dynamics of individual trees and thus allow for the scaling of tree‐scale processes to large areas (Seidl et al., 2012). The model explicitly simulates tree competition for resources such as light, water, and nutrients. A light use efficiency approach (Landsberg & Waring, 1997) is used to simulate the production physiology. Carbon starvation is used as a process‐oriented indicator of tree stress, which can result from competition for resources as well as suboptimal environmental conditions for tree growth (e.g., drought).

iLand's mechanistic representation of forest disturbances and vegetation dynamics, as well as the climatic sensitivity of these processes, makes it well suited for the research of disturbance dynamics under climate change (e.g., Dobor et al., 2018; Seidl & Rammer, 2017; Seidl, Rammer, & Blennow, 2014). Wind disturbances are initiated by the wind speed of severe wind events provided as an external input to the simulation. The wind impact is simulated iteratively, with the forest structure (including the appearance of new edges) being updated after each iteration in the event of breakage or windthrow (Seidl, Rammer, et al., 2014; Supplement A). The implementation of bark beetle disturbances considers bark beetle phenology and development, spatially explicit dispersal of beetles, colonization, and tree defense, as well as temperature‐related overwintering success (Seidl & Rammer, 2017). Outbreaks are typically triggered by wind disturbance; salvage removal of windfelled trees can therefore be applied to reduce the outbreaks (Dobor et al., 2020). A detailed description of the implementation of wind and bark beetle disturbance in iLand is provided in Supplement A.

Flexible implementation of management operations, which include planting after harvests or natural disturbances, thinning, harvesting, and postdisturbance salvaging, allows for testing the effects of various disturbance management strategies (Dobor et al., 2019, 2020; Honkaniemi et al., 2020). iLand integrates an agent‐based model of forest management (Rammer & Seidl, 2015), in which general stand treatment programs (i.e., a sequence of management interventions applied over the course of stand development) are dynamically adapted to the forest state emerging from the simulation. These features allow for testing the efficiency of measures taken in response to the simulated disturbance, considering the dynamically changing vegetation structure. The tested management interventions are implemented in the model as follows:

- Planting is applied after harvests and stand‐replacing disturbances that affected a prescribed level of growing stock (75% in this study). Planting is based on prescriptions defining details of tree species, seedling dimensions, and spacing between the seedlings. Planting prescriptions can differ between stands, depending on site conditions or management objectives. The already established regeneration can be kept or removed.
- Thinning and harvesting are applied based on prescribed timing and intensity of removal. Different criteria on tree removal can be defined to implement practices such as clearcutting, shelterwood, or selection cut. Each stand has a stand treatment program assigned that defines the sequence of interventions.
- Salvaging is applied to harvest timber affected by disturbances. Different intensities of salvage removal can be prescribed, affecting forest carbon stocks, dynamics of secondary disturbances, and the deadwood patterns. The incidence of disturbances and subsequent salvage logging supersede regular management operations, resetting the default stand treatment program.

The model was extensively tested across a range of ecosystems in Europe and North America in previous studies (Honkaniemi et al., 2020; Silva Pedro et al., 2015; Thom et al., 2017). A detailed evaluation of simulated productivity, natural mortality, and regeneration patterns for the landscape studied here was conducted by Dobor et al. (2018). All testing exercises conducted for Central Europe proved good ability of the model to simulate ecosystem dynamics in this environment.

### 2.3 Basic simulation setup

Prior to scenario simulations, an 800‐year spin‐up run was performed to estimate the initial litter, dead wood, and soil C pools. The spin‐up run was also used to initialize stand structures in a manner consistent with the internal logic of the model. The procedure used assimilates information on the current composition and structure of forest stands (here based on forest management plan records; see Supplement B) to ensure that the resulting initial forest state for simulation is consistent with the model internal logic and represents the current structure and composition of the forest (see Thom et al., 2018 for details).

The scenario simulations were run for 100 years starting from the end‐conditions of the preceding spin‐up run. Each simulation was driven by five climatic scenarios (reference climate and four projected climates, see Supplement C). Each simulated forest development was exposed to five prescribed wind events, with parameters sampled from the distribution parameterized based on past meteorological observations for the area. The intensity of events was set to reach the average annual amount of disturbed trees recorded in the national forest disturbance statistics for the period 1990–2010, which range between 0.9 and 2.2 m^3^ ha^−1^ year^−1^ (Dobor et al., 2020; Konôpka et al., 2016). Each scenario run was replicated 10 times to account for the stochasticity in the simulations. The value of the so‐called background infestation probability (a parameter related to bark beetle disturbance (Seidl & Rammer, 2017) was varied between the replicates in a range of 0.0005–0.0025 (Honkaniemi et al., 2020).

The implemented baseline management included tending, thinning, and harvesting, with timing and intensity of operations modeled after the management practice currently applied in the region (Halaj & Petráš, 1998). Depending on the site, 3–4 thinning operations were applied and rotation periods ranged from 90 to 140 years. In spruce monocultures, clearcut management was applied, whereas shelterwood management was simulated in mixed stands.

The simulations were run under the conditions defined by two regional climate models (RCM) driven by two Representative Concentration Pathway scenarios (RCP4.5 and RCP8.5). The RCMs were selected to represent the variability of climate change signal emerging from the large ensemble of climate projections developed in the frame of the CORDEX project (Giorgi et al., 2009) (Supplement C). A reference climate series was generated by randomly sampling years with replacement from the period 1996–2016.

### 2.4 Disturbance management experiment

The previously described baseline management was modified to accommodate different combinations and settings of disturbance management actions. These include (a) targeted change in tree species composition via planting on cleared areas to reduce the overall forest vulnerability, (b) instant removal of windfelled trees, which can trigger or reinforce the outbreak of bark beetles, and (c) reduction of forest rotation length to reduce the proportion of mature trees, which are susceptible to both bark beetle and wind disturbance.

We organized these measures around two management narratives that are being intensively discussed in the Central European production forestry; (a) the industry demand‐driven effort to maintain high proportions of Norway spruce in the forest, and (b) efforts to adapt the tree species composition to climate change and intensified disturbances via recovering natural species composition, which has been markedly altered over the last two centuries in many production forests in Central Europe (Klimo et al., 2000; Spiecker et al., 2004).

To address these two objectives, we implemented two seedling planting schemes on cleared areas. The first one promoted Norway spruce in species composition; depending on site, its share ranged from 50% to 70% (natural regeneration was, however, acting concurrently). The second one promoted site‐suitable tree species following the natural species composition of the forest (after Rizman et al., 2005), which predominantly consisted of European beech, Silver fir, European larch, and pine. The share of Norway spruce did not exceed 20% in this planting variant.

We combined each of these planting variants with salvage removal of windfelled trees and the rotation length reduction. The salvaging was applied with 90% intensity, which was found by Dobor et al. (2019) to be the minimal intensity required to dampen the simulated outbreak of bark beetles. At the same time, such intensity represents a realistic approximation of the applied management practice. We simulated the reduction of the rotation length by 40% relative to the currently applied rotation (100 years for spruce stands, and 115 years for broadleaved species, on average). The rotation length was, however, not allowed to be <60 years. This level of reduction still conforms with the criteria for the production of softwood timber and can be expected to dampen the disturbance dynamics to a certain extent.

The disturbance reduction effect of different management actions was assessed by comparing the level of disturbed growing stock (m^3^ ha^−1^ year^−1^, average over the simulation period) reached under management variants B1 to C4 (Table 1) against the reference management A, which did not contain any disturbance management action. The total number of simulation runs in this experiment was as follows: 8 managements × 5 climates (four climate change scenarios and the reference climate) × 10 replicates, that is, 400 simulations.

**TABLE 1:** Tested combinations of disturbance management measures

Code	Management narrative	Planting scheme	Disturbance reduction actions		
			Change in species composition	Salvaging	Rotation length reduction
A	Reference management, focus on spruce timber production, no disturbance management action taken	Dominance of spruce seedlings (50%–70%, depending on site)	0	0	0
B1	A + reducing risk of disturbance via high‐intensity salvaging		0	1	0
B2	A + reducing risk of disturbance via rotation length reduction		0	0	1
B3	A + reducing risk of disturbance via rotation length reduction and high‐intensity salvaging		0	1	1
C1	Focus on adaptive change in species composition via planting on disturbed and harvested sites	Dominance of seedlings of nonspruce site‐matching species (more than 80%, depending on site)	1	0	0
C2	C1 + reducing risk of disturbance via high‐intensity salvaging		1	1	0
C3	C1 + reducing risk of disturbance via rotation length reduction		1	0	1
C4	C1 + reducing risk of disturbance via high‐intensity salvaging and rotation length reduction		1	1	1

## 3 RESULTS

### 3.1 Simulated disturbance patterns

**FIGURE 2:**
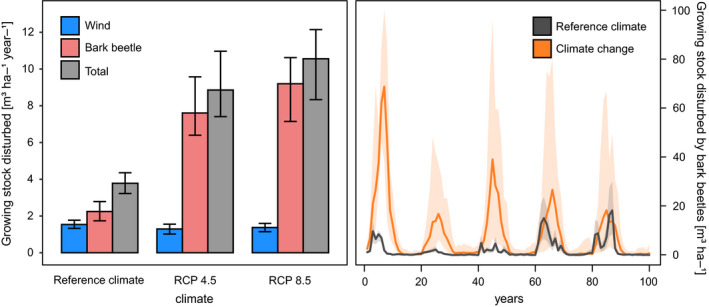
Growing stock disturbed by wind and bark beetles during the 100‐year simulation period under reference climate and two groups of climate change scenarios driven by RCP4.5 and RCP8.5 (a). Medians and 10%–90% quantiles calculated from 10 replicate simulations, 8 management regimes, and 4 climate change scenarios are shown. (b) The temporal development of growing stock disturbed by bark beetles under the reference climate (averaged over managements and replicates) and climate change (averaged over managements, RCP scenarios, and replicates)

The average level of wind disturbed growing stock simulated under the reference climate was 1.2–1.8 m^3^ ha^−1^ year^−1^ (range of eight management systems and 10 replicates), which falls into the observed range of 0.9–2.2 m^3^ ha^−1^ year^−1^. Under climate change, wind disturbance decreased by 11%–16% relative to the reference climate, which likely accounts for the competing interaction between wind and bark beetles (Figure 2a).

Each wind event triggered a multi‐year bark beetle outbreak of varying size, depending on the actual amount of windfelled trees, weather, and stand conditions (Figure 2b; Supplement D). Under reference climate, disturbed growing stock was 1.6–3.0 m^3^ ha^−1^ year^−1^, that is, slightly exceeded the growing stock disturbed by the triggering windthrows. Climate change produced a strong amplifying effect on the outbreaks, and the level of growing stock disturbed exceeded the reference value by 239% under RCP4.5 and 310% under RCP8.5 (median increase, Figure 2).

### 3.2 Disturbance management performance

**FIGURE 3:**
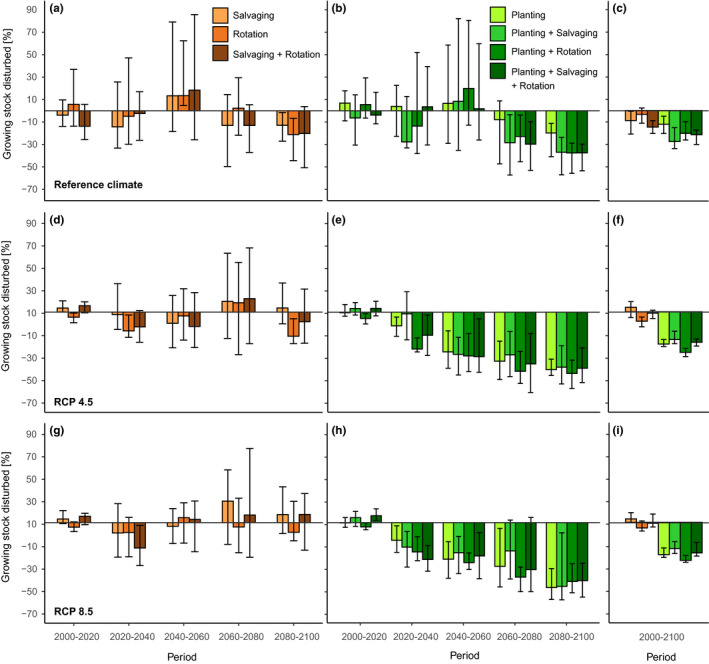
Relative differences between the level of growing stock disturbed by wind and bark beetles under management systems containing different combinations of disturbance management actions, and the baseline management, with no disturbance management actions. Management systems on the left (brownish) promote planting of spruce on disturbed and harvested stands, whereas systems on the right (greenish) promote adaptive changes in species composition by planting less vulnerable site‐matching tree species

Under the reference climate, the total level of growing stock disturbed was substantially reduced by different combinations of management measures (Figure 3a,b). In management systems promoting spruce in species composition, however, the mitigation effect highly varied within (i.e., the interreplicate variation) and between treatments. Still, the average reduction effect over the simulation period was 8% for salvaging, 3% for rotation length reduction, and 13% for the combination of latter two measures (separate effects on wind and bark beetle disturbance are provided in Supplement E).

Management systems promoting adaptive changes in tree species composition more efficiently reduced the disturbance than the previous systems, though lead times were long (from ca. 2060) (Figure 2b). The simultaneous application of different treatments amplified the disturbance reduction effect. Systems containing salvaging (C2 and C4) were most efficient, reducing the disturbance by 19%–25% relative to the reference treatment A (Figure 3c).

Climate change markedly altered patterns identified under the reference climate. Disturbance treatments were inefficient in reducing disturbance impacts in management systems promoting spruce (Figure 2d,g). On the other hand, climate change amplified the efficiency of disturbance treatments in systems promoting adaptive changes in tree species composition. These measures started to be effective much earlier than under the reference climate; significant disturbance reduction started to be observed as soon as 2020. The combination of different measures did not significantly increase the disturbance reduction effect of changing tree species composition, particularly in the second half of the simulation period.

### 3.3 Underlying changes in forest structure

**FIGURE 4:**
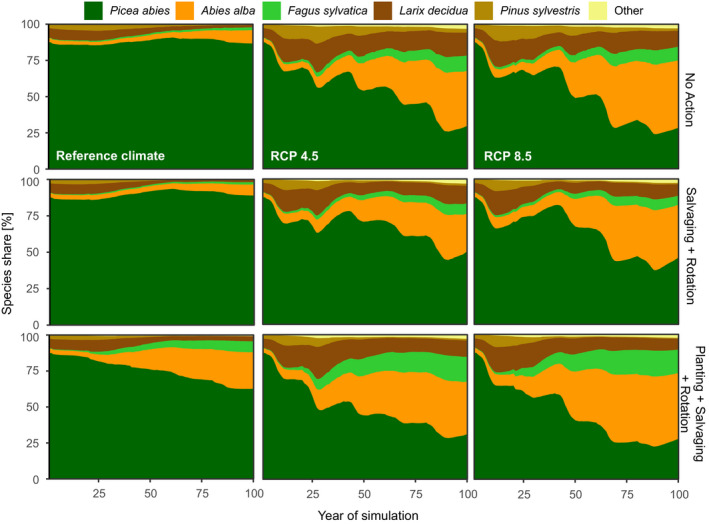
Development of tree species composition in the study landscape under different management and climate change scenarios. The upper row is the baseline management, which included no disturbance management actions and promoted spruce in planting on cleared areas. The middle row reflects an alternative to the baseline management, with salvage removal and rotation length reduction applied. The bottom row reflects the most proactive management, which promotes nonspruce species in planting, with intensive salvaging of windfelled trees and a reduced rotation length

The tested management interventions affected forest susceptibility to disturbance mainly via changes in forest age structure and species composition. The initially high proportion of Norway spruce persisted under the reference climate, when the level of disturbance was low (Figure 4). This persistence was supported by the dominance of spruce in planting. The modified planting scheme mainly caused the proportion of Silver fir and European beech to increase, while spruce remained dominant. Even a moderate climate change (RCP4.5) caused spruce to decline sharply throughout the simulation period, and this decline was further amplified by the change in planting. The main replacement species were Silver fir, European beech, and European larch. Information about the remaining management variants is provided in Supplement F.

**FIGURE 5:**
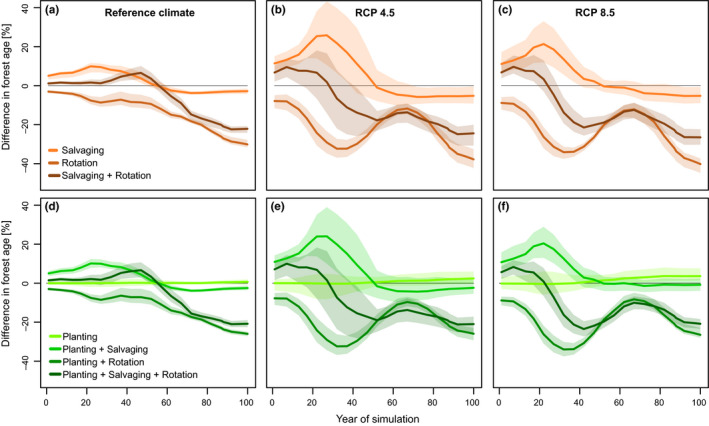
Differences between the mean forest age on the study landscape simulated under management systems involving different combinations of disturbance reduction measures and the reference management system, where no measures were applied. The upper row indicates management systems with a dominance of spruce in planting after harvests and disturbances. The bottom row indicates management systems promoting site‐matching tree species

Forest age sharply decreased under management systems involving the reduction of rotation length compared to the reference management A (Figure 5). Whereas the decrease was gradual under reference climate, age fluctuation was more erratic under climate change and age reduction occurred faster. The reduction reached −20 to −30% of the initial forest age.

## 4 DISCUSSION

Previous studies indicated that Norway spruce forests may not be sustained in many regions of Europe because of intensifying outbreaks of bark beetles, genetic maladaptation to future climates, and sensitivity to climatic stress (Frank et al., 2017; Marini et al., 2012; Seidl, Schelhaas, et al., 2014; Zang et al., 2014). The role of active management of forests disturbances, however, has not been included in these investigations although it is an integral aspect of European forest management (Berryman, 1988; Wermelinger, 2004). We showed that contrast in the vulnerability of monospecific forests and forests managed for diversity is considerably amplified by climate change. We also found that management measures that were successfully applied in the past are becoming inefficient under warmer climate amplifying the disturbance dynamics, which particularly applies to the forests dominated by Norway spruce.

### 4.1 Implications for ecosystem management

We found that the studied ecosystem was relatively stable under past climate and the level of disturbance was low. Such dynamics agree with the national forest damage statistics (e.g., Gubka et al., 2013), which indicated that the level of disturbance was quite low before 1995. This suggests that intensively applied measures (planting, sanitary operations, etc.) managed to sustain the forest despite its structure and resilience being compromised by the previous long‐term production‐oriented management. In our study, these conditions correspond with simulation designs A and B1‐B4 exposed to the reference climate.

Exposing the spruce dominated system to climate change increased the disturbance intensity by 140%–172% and caused the proportion of Norway spruce to decline sharply. Parallels can be drawn between this simulated development and the recently observed collapse of spruce forests in some regions of Europe—Central Europe being an epicenter—triggered by climate extremes and large‐scale outbreaks of bark beetles (Hlásny et al., 2019; Senf & Seidl, 2018). Moreover, we found that this increase in disturbance intensity cannot be controlled by the here tested management measures, despite the measures being applied with a high intensity (90% removal of windfelled trees and 40% reduction of the rotation length). This is a striking difference from the disturbance management applied under past climate, where disturbance intensity was lower and it could have been further reduced by management. Conversely, we found that the forest managed for diversity showed lower disturbance rates even without applying any other measures (i.e., without the salvage removals and rotation length reduction). These findings provide a new perspective on the role of adaptive changes in species composition in disturbance management and can clarify some misconceptions about the transferability of past management tactics to the qualitatively new conditions produced by climate change.

Consistent with previous studies, we found that the change in tree species composition toward a higher proportion of less vulnerable and site‐adapted species has paramount importance in managing forests under climate change (Jandl et al., 2019). Diverse ecosystems generally show lower disturbance rates compared with monospecific forests (Griess et al., 2012; Neuner et al., 2015) and are also superior in the provisioning of ecosystem services (Mori, 2017). Still, some previous studies suggested that the pest control effect may depend more on species composition of the forest than on diversity (Koricheva et al., 2006). Accordingly, the here presented disturbance mitigation effect needs to be considered as a function of both replacement of vulnerable Norway spruce by other tree species and the increase in stand‐ and landscape‐scale diversity, which, for example, dilutes the host trees and prevents the large‐scale spread of bark beetles (Honkaniemi et al., 2020; Silva Pedro et al., 2015).

We found that the same disturbance reduction effect can be reached by applying different management actions. This finding deserves recognition in forestry practice, because measures such as salvaging, modifying harvesting regimes or changing tree species composition affect ecosystem dynamics, and provision of ecosystem services in different ways (Leverkus, Benayas, et al., 2018; Roberge et al., 2016). Quantitative understanding of these measures thus allows formulating management strategies consistent with local management objectives. Notably, measures such as salvage removal of windfelled trees and rotation length reduction did not significantly amplify the disturbance reduction effect produced by mere change in species composition. This indicates that these measures could be potentially avoided, providing multiple benefits for forestry economies and natural ecosystem dynamics. For example, maintaining older conditions on the landscape (i.e., avoiding rotation length reduction) can be beneficial from the viewpoints of biodiversity, forest carbon, and landscape scenic values (Roberge et al., 2016; Thom et al., 2019). Maintaining deadwood in the forest (i.e., avoiding or reducing salvage removals) supports water and climate regulation functions, increases forest diversity, including pests’ antagonists, and preserves deadwood carbon stocks (Lassauce et al., 2011). Therefore, complex considerations are needed to formulate a proper combination of disturbance management actions to reach the desired control over the disturbance dynamics and not to compromise important ecosystem services.

### 4.2 Methodological aspects and limitations

Here, we used a highly complex simulation model to investigate the interactions between disturbance dynamics, management, vegetation feedbacks, and climate change. Although such an approach allowed identifying and attributing the effects of different management actions, the uncertainty related to the representation of individual processes and model assumptions needs to be carefully considered (Huber et al., 2020). Although the model's use is supported by numerous testing exercises that particularly addressed forest productivity, regeneration, and natural mortality (e.g., Dobor et al., 2018), reproducing complex disturbance regimes in models remains challenging (Seidl et al., 2011). High stochasticity of disturbance events complicates testing the simulation outputs against the observed impacts (but see for example. Seidl & Rammer, 2017). We here prescribed the intensity of wind impacts to match the long‐term observations, whereas the simulated windthrow pattern and the interaction with bark beetle dynamics were simulated as emergent properties of the used simulation framework. More comprehensive testing of the simulated disturbance patterns against observation would provide useful support to the presented findings.

Given the high complexity of our experimental design, we only investigated one level or salvaging intensity and rotation length reduction, though management practice may require more complex information (see e.g., Dobor et al., 2019, 2020; Zimová et al., 2020). The tested intensities were, however, near to the logistic limits of the current forest management and can thus be interpreted as the reachable maximum under the operational management conditions.

The complexity of our design can be further increased by including other management variants, which stress, for example, adaptive changes in species composition, including altitudinal shift of zonal trees species (Moser et al., 2010) and introduction of species that do not participate in the actual species composition. In the Central European forestry, these species may include, for example, native oak species (*Quercus* sp.) which are expanding their ranges under climate change (e.g., Mette et al., 2013) as well as introduced species of which the Douglas fir (*Pseudotsuga menziensii*) has received considerable attention (Spiecker et al., 2019). Although such changes would not directly affect the disturbance dynamics in the current modeling framework (their vulnerability is similar to other species on the study landscape), they could indirectly affect the disturbance dynamics via different rates of establishment on disturbed sites.

## 5 CONCLUSIONS

Management of wind and bark beetle disturbances constitutes an integral part of European forestry; yet, many approaches are based on plausible or intuitive narratives rather than on tested and data‐driven concepts. We presented a new perspective on disturbance management in the Central European production forests and particularly on the interactions of adaptive changes in species composition with other management measures. Consistent with previous studies, we found a contrasting sensitivity of monospecific and species‐diverse forests to disturbance impacts. However, we showed that climate change further amplifies this contrast and favors management fostering forest diversity, which can exert better control over disturbance dynamics even without pervasive measures compromising the biodiversity and resilience of the forest. These findings can justify some misconceptions about disturbance management under climate change and can support the formulation of improved management strategies.

## CONFLICT OF INTEREST

None declared.

## AUTHOR CONTRIBUTION

**Laura Dobor:** Formal analysis (equal); Methodology (equal); Software (equal); Visualization (equal); Writing‐review & editing (equal). **Tomáš Hlásny:** Conceptualization (lead); Formal analysis (equal); Funding acquisition (lead); Project administration (lead); Writing‐original draft (lead); Writing‐review & editing (lead). **Soňa Zimová:** Data curation (supporting); Formal analysis (equal); Resources (equal); Software (supporting); Writing‐review & editing (supporting).

## Supporting information

Supplementary Material

## Data Availability

Results are archived in the Zenodo open‐access repository, http://doi.org/10.5281/zenodo.4020390. Additional information on the used ecosystem model, including the source code can be found at http://iland.boku.ac.at.
