# Supplementary material for: Contrasting vulnerability of monospecific and species‐diverse forests to wind and bark beetle disturbance: The role of management
Source: Ecol Evol. 2020 Oct 16;10(21):12233–45. doi: 10.1002/ece3.6854 (PMC7663067; doi:10.1002/ece3.6854)
Supplement: Supplementary file 1 — Supplementary Material [file ECE3-10-12233-s001.docx]

**Supplementary material**

**Contrasting vulnerability of monospecific and species diverse forests to natural disturbances: The role of management**

Laura Dobor, Tomáš Hlásny**,* Soňa Zimová

*****Corresponding author:** Tomáš Hlásny, Czech University of Life Sciences Prague, Faculty of Forestry and Wood Sciences, Kamýcká 129, 165 21 Prague 6, Czech Republic; e-mail: hlasny@fld.czu.cz, phone: +421 905 708 539

**Supplement A: Implementation of wind and bark beetle disturbance in the model iLand**

Wind disturbances are initiated by the wind speed of severe wind events provided as external input to the simulation. The model initiates wind disturbances in locations, where canopy rugosity changes abruptly, i.e., where vertical differences between the top heights of neighbouring grid cells exceed 10 m (Blennow and Sallnäs, 2004). Next, wind speed at the canopy top height is calculated based on a vertical wind profile at the stand edge (Gardiner et al., 2000), and individual-tree turning coefficients (Hale et al., 2012) are calculated. The latter two information are used to calculate the critical wind speeds for uprooting and tree breakage based on the approach of Gardiner et al. (2000). If the soil is frozen, only the stem breakage is allowed to occur. The final evaluation of the impact of wind on forest is based on comparison of the prevailing wind speed to the critical windspeed; if the critical wind speed is exceeded, the tree is broken or uprooted. The disturbance impact is simulated iteratively, with forest structure (including the appearance of new edges) being updated after each iteration if breakage or windthrow was simulated.

The process-based implementation of bark beetle disturbances considers bark beetle phenology and development, spatially explicit dispersal of beetles, colonization and tree defence, as well as temperature-related overwintering success (Seidl and Rammer, 2017). Large outbreaks are typically triggered by wind disturbance, but smaller outbreaks occur also independently based on a climate-sensitive background probability. Bark beetle development is simulated based on the beetle phenology model by Baier et al. (2007). The model tracks beetle cohorts rather than individuals, with a cohort being defined as the minimum number of beetles needed to successfully colonize a tree. Every brood tree disperses a number of beetle cohorts determined by the reproductive rate of the beetle (Wermelinger and Seifert, 1999). Attacking beetle cohorts need to first overcome the defence system of the tree, which is approximated by its dynamically simulated non-structural carbohydrate reserves. The tree can be attacked in multiple waves of beetle cohorts in one vegetation period if the climate allows for the development of multiple beetle generations per years.

**References**

Baier, P., Pennerstorfer, J., Schopf, A., 2007. PHENIPS-A comprehensive phenology model of Ips typographus (L.) (Col., Scolytinae) as a tool for hazard rating of bark beetle infestation. Forest Ecology and Management 249, 171–186. https://doi.org/10.1016/j.foreco.2007.05.020

Blennow, K., Sallnäs, O., 2004. WINDA - A system of models for assessing the probability of wind damage to forest stands within a landscape. Ecological Modelling 175, 87–99. https://doi.org/10.1016/j.ecolmodel.2003.10.009

Gardiner, B., Peltola, H., Kellomäki, S., 2000. Comparison of two models for predicting the critical wind speeds required to damage coniferous trees. Ecological Modelling 129, 1–23. https://doi.org/10.1016/S0304-3800(00)00220-9

Hale, S.E., Gardiner, B.A., Wellpott, A., Nicoll, B.C., Achim, A., 2012. Wind loading of trees: Influence of tree size and competition. European Journal of Forest Research 131, 203–217. https://doi.org/10.1007/s10342-010-0448-2

Seidl, R., Rammer, W., 2017. Climate change amplifies the interactions between wind and bark beetle disturbances in forest landscapes. Landscape Ecology 32, 1485–1498. https://doi.org/10.1007/s10980-016-0396-4

Wermelinger, B., Seifert, M., 1999. Temperature dependent reproduction of the spruce bark beetle Ips typographus, and analysis of the potential population growth. Ecological Entomology 24, 103–110.

**Supplement B: Stand, site and climate data**

Data used to initialize trees on the landscape were taken from forest management plans (FMP) provided by the National Forest Centre (NFC), Slovakia. Information on soil depth and plant available nutrient were taken from the forest soil database supervised by the NFC too. The source data contained a relative nutrient content only (0-1), which was used to estimate the plant-available nitrogen (kg m^-2^ year^-1^) based on iLand-internal model logic (Seidl, Rammer, et al., 2012).

Data for all climate scenarios were interpolated to a 100m grid from a single RCM grid cell located within the landscape using the MTClim model (Mountain Microclimate Simulation Model, Hungerford et al., 1989). MTClim was also used to derive daily global radiation data. CO_2_ concentrations used to drive the forest simulations were defined by the two RCP scenarios used, and reached 538 ppm and 936 ppm in 2100 under RCP4.5 and RCP8.5 runs, respectively (see also Dobor et al., 2018).

**References**

Seidl, R., Rammer, W., Scheller, R. M., & Spies, T. A. (2012). An individual-based process model to simulate landscape-scale forest ecosystem dynamics. Ecological Modelling, 231, 87–100. doi:10.1016/j.ecolmodel.2012.02.015

Hungerford, R. D., Nemani, R. R., Running, S. W., & Coughlan, J. C. (1989). MTCLIM: A mountain microclimate simulation model. USDA Forest Service Res. Paper, 52.

Dobor, L., Hlásny, T., Rammer, W., Barka, I., Trombik, J., Pavlenda, P., … Seidl, R. (2018). Post-disturbance recovery of forest carbon in a temperate forest landscape under climate change. Agricultural and Forest Meteorology, 263, 308–322. doi:10.1016/j.agrformet.2018.08.028

**Supplement C: Used climate model results and projected climate change**

Table C1. Description of the available combinations of global and regional climate models

|  | **Global Climate Model** | | **Regional Climate Model** | | |
| --- | --- | --- | --- | --- | --- |
| 1 | CM5A-MR | Institut Pierre-Simon Laplace, France (IPSL) | RCA4 | Swedish Meteorological and Hydrological Institute, Rossby Centre, Sweden (SMHI) | Strandberg et al., 2014 |
| 2 | CNRM-CM5 | Météo-France / Centre National de Recherches Météorologiques, France (CNRM) | RCA4 | Swedish Meteorological and Hydrological Institute, Rossby Centre, Sweden (SMHI) | Strandberg et al., 2014 |
| 3 | EC-EARTH | Irish Centre for High-End Computing (ICHEC) | HIRHAM5 | Danish Meteorological Institute, Denmark (DMI) | Christensen et al., 2007 |
| 4 | EC-EARTH | Irish Centre for High-End Computing (ICHEC) | RACMO22E | Royal Netherlands Meteorological Institute, De Bilt, The Netherlands (KNMI) | van Meijgaard et al., 2008 |
| 5 | EC-EARTH | Irish Centre for High-End Computing (ICHEC) | RCA4 | Swedish Meteorological and Hydrological Institute, Rossby Centre, Sweden (SMHI) | Strandberg et al., 2014 |
| 6 | MOHC-HADGEM2-ES | Met Office Hadley Centre, United Kingdom (MOHC) | RCA4 | Swedish Meteorological and Hydrological Institute, Rossby Centre, Sweden (SMHI) | Strandberg et al.,2014 |
| 7 | MPI-ESM-LR | Max Planck Institute for Meteorology, Germany (MPI) | RCA4 | Swedish Meteorological and Hydrological Institute, Rossby Centre, Sweden (SMHI) | Strandberg et al.,2014 |

Table C2. Projected changes of temperature and precipitation in the growing season (April-September) for periods 2031-2060 and 2071-2100 based on six climate models and two RCP scenarios compared to the period 1996-2016. The last row shows average projected temperature or precipitation for both periods and RCP scenarios.

|  | Expected changes for 2031-2060 | | | | Expected changes for 2071-2100 | | | |
| --- | --- | --- | --- | --- | --- | --- | --- | --- |
| Model | Temperature (IV-IX) [°C] | | Precipitation (IV-IX) [%] | | Temperature (IV-IX) [°C] | | Precipitation (IV-IX) [%] | |
| RCP | RCP4.5 | RCP8.5 | RCP4.5 | RCP8.5 | RCP4.5 | RCP8.5 | RCP4.5 | RCP8.5 |
| 1 | 0.9 | 1.5 | -18.0 | -10.3 | 1.9 | 4.0 | -22.3 | -21.2 |
| 2 | 0.2 | 0.3 | -21.6 | -7.2 | 1.1 | 2.7 | -16.6 | -15.5 |
| 3 | 0.9 | 1.0 | -12.8 | -4.1 | 0.9 | 2.7 | 4.2 | 3.7 |
| 4 | 0.2 | 0.7 | -3.4 | -5.0 | 1.0 | 2.7 | -2.9 | -8.7 |
| 5 | 0.8 | 1.4 | -22.9 | -16.7 | 1.6 | 3.6 | -14.9 | -24.0 |
| 6 | 1.1 | 1.7 | -13.9 | -15.4 | 2.1 | 4.1 | -15.1 | -22.7 |
| 7 | 0.7 | 1.1 | -18.1 | -7.4 | 1.1 | 3.3 | -21.8 | -19.9 |
| AVG | 0.7 | 1.1 | -15.8 | -9.4 | 1.4 | 3.3 | -12.8 | -15.5 |


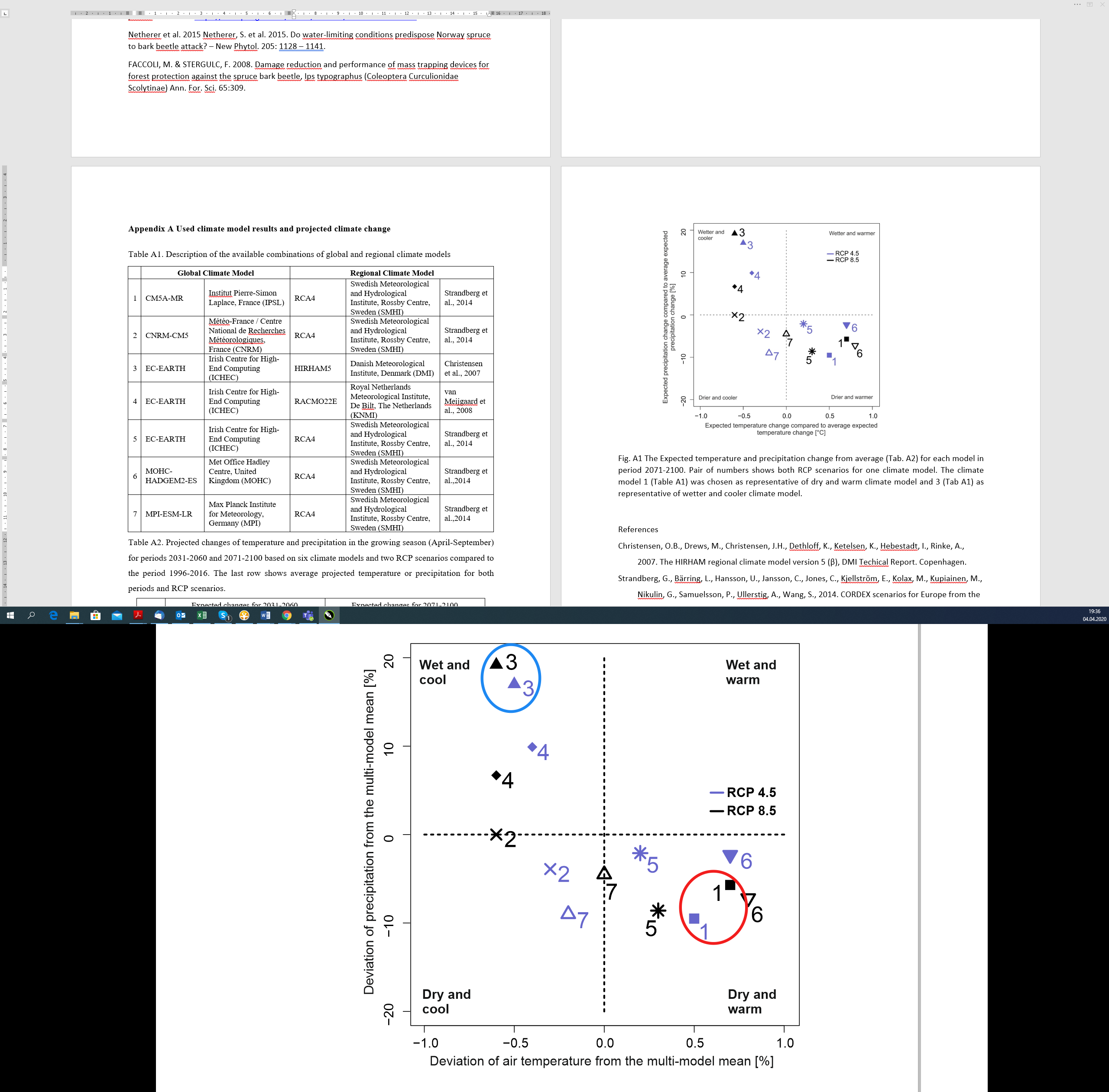


Fig. C1 Deviations of mean annual air temperature and precipitation totals projected by seven climate models driven by two RCP scenarios from the all-model mean values (see model codes in Table C2). The data are for the period 2071-2100. The circles indicate two contrasting climate projections, which were used in the current study.

**References**

Christensen, O.B., Drews, M., Christensen, J.H., Dethloff, K., Ketelsen, K., Hebestadt, I., Rinke, A., 2007. The HIRHAM regional climate model version 5 (β), DMI Techical Report. Copenhagen.

Strandberg, G., Bärring, L., Hansson, U., Jansson, C., Jones, C., Kjellström, E., Kolax, M., Kupiainen, M., Nikulin, G., Samuelsson, P., Ullerstig, A., Wang, S., 2014. CORDEX scenarios for Europe from the Rossby Centre regional climate model RCA4. SMHI Rep. Meteorol. Climatol. 116, 1–45.

van Meijgaard, E., van Ulft, L.H., van de Berg, W.J., Bosveld, F.C., van den Hurk, B., Lenderink, G., Siebesma, A.P., 2008. Technical Report 302: The KNMI regional atmospheric climate model RACMO version 2.1. De Bilt.


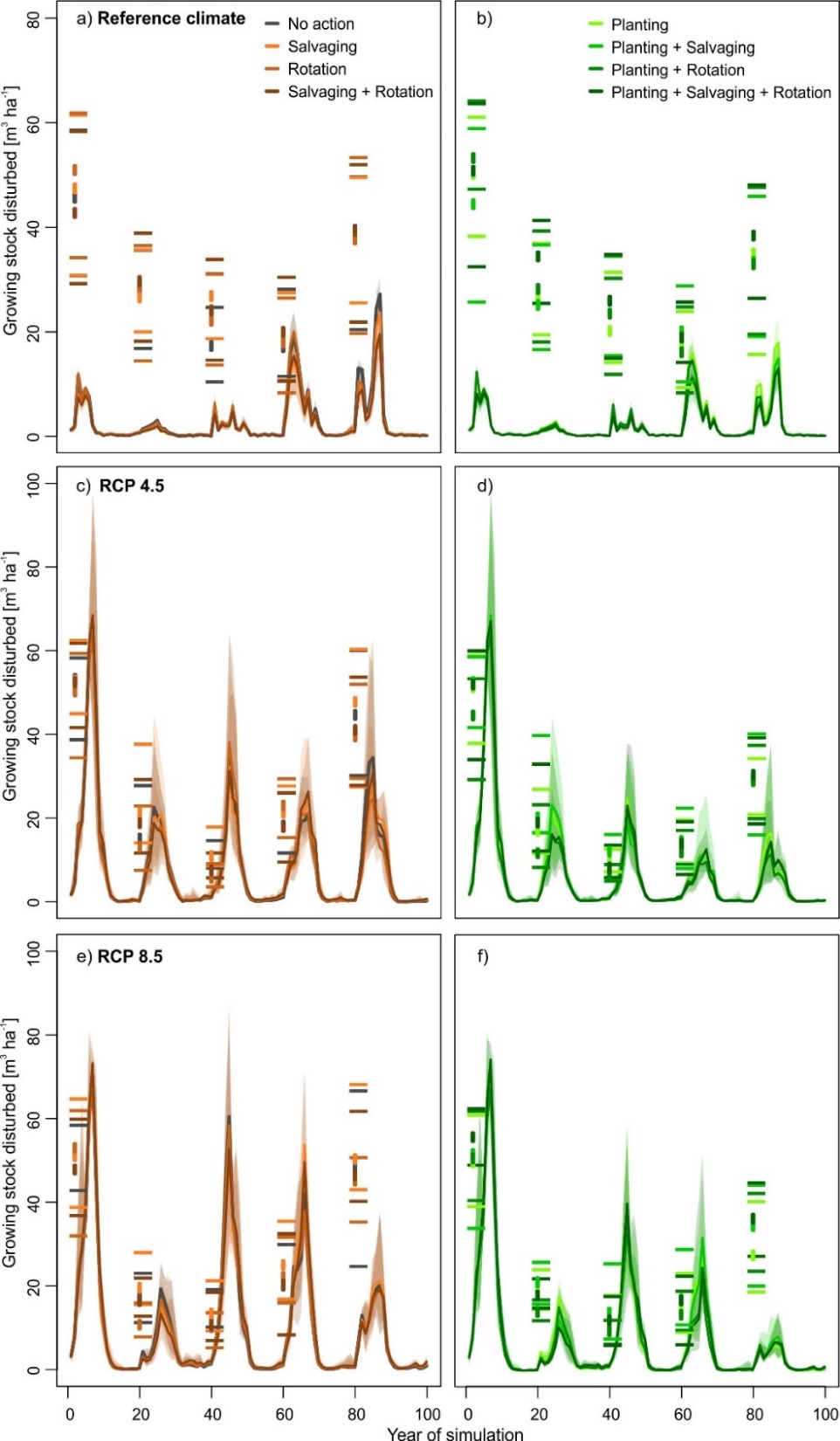
**Supplement D: Time series of wind and bark beetle disturbance different climates and management regimes.**

Fig. D1 Time series of wind and bark beetle disturbance under different climates and management regimes. Lines indicate bark beetle outbreaks and symbols windthrows. Managements description is in Table 1 in the text.

**Supplement E: Disturbance reduction effects of different management actions**


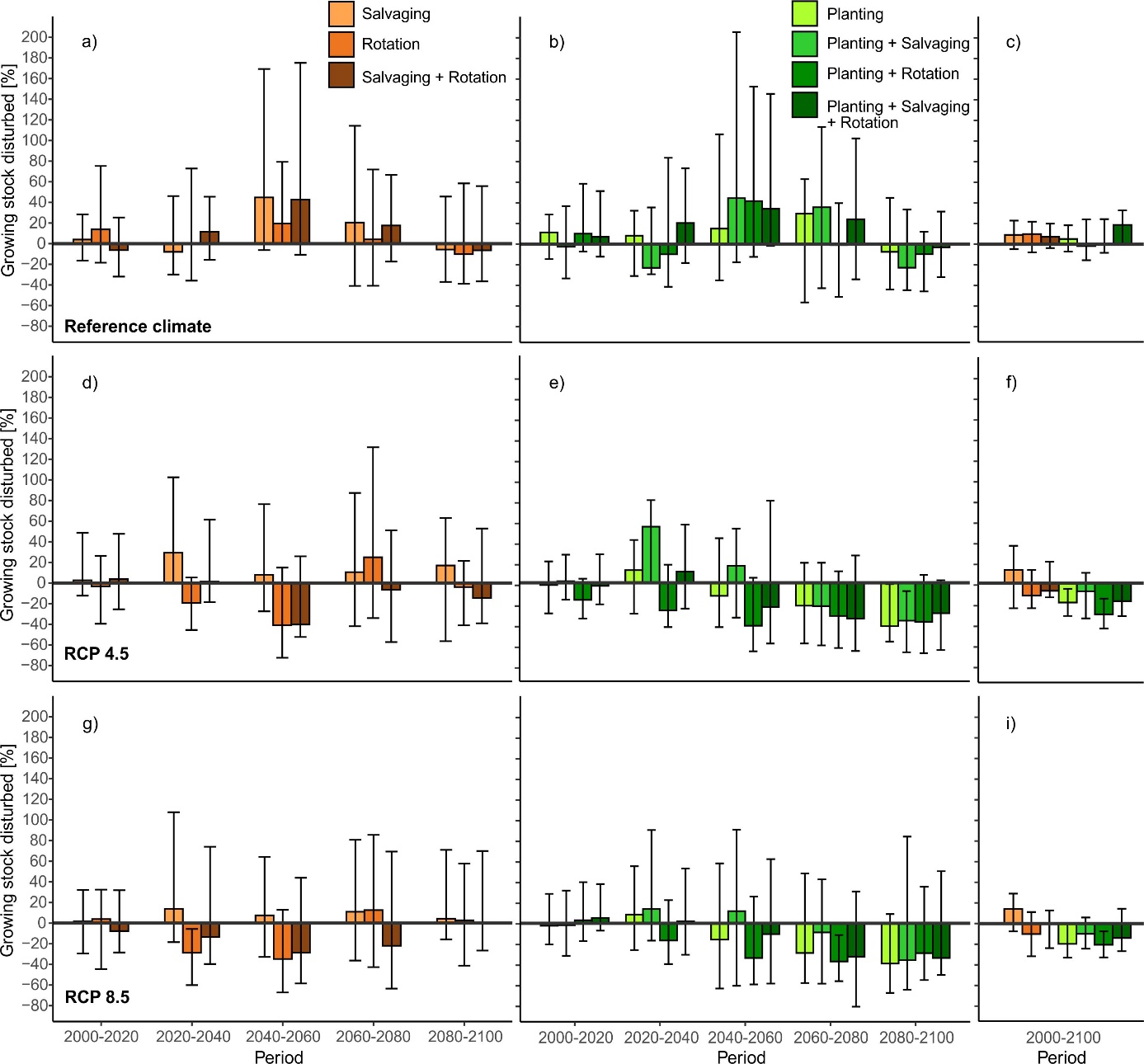


Fig. E1 Effect of different management regimes on wind disturbance under different climates. Managements description is in Table 1 in the text.


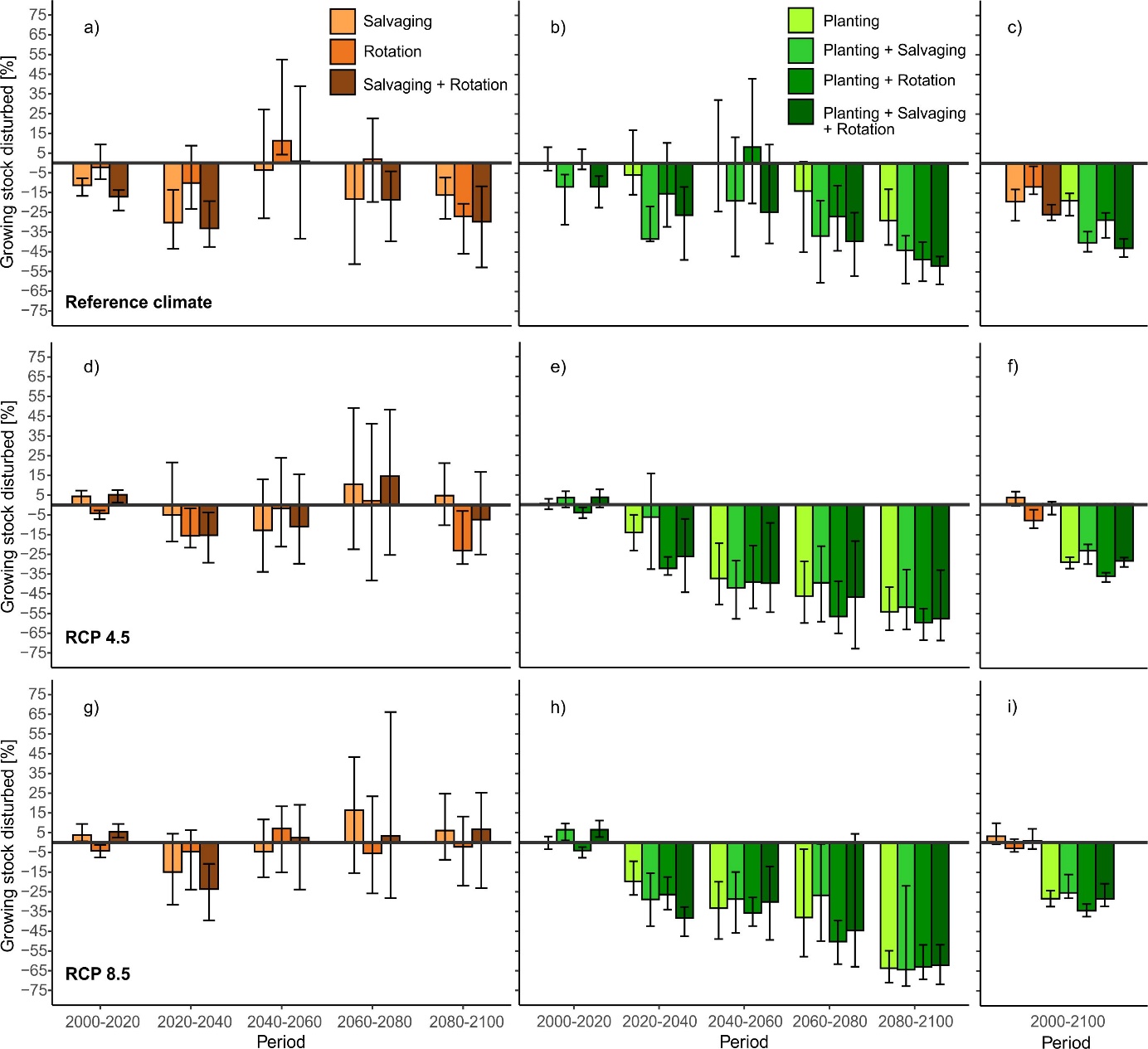


Fig. E2 Effect of different management regimes on bark beetle disturbance under different climates. Managements description is in Table 1 in the text.


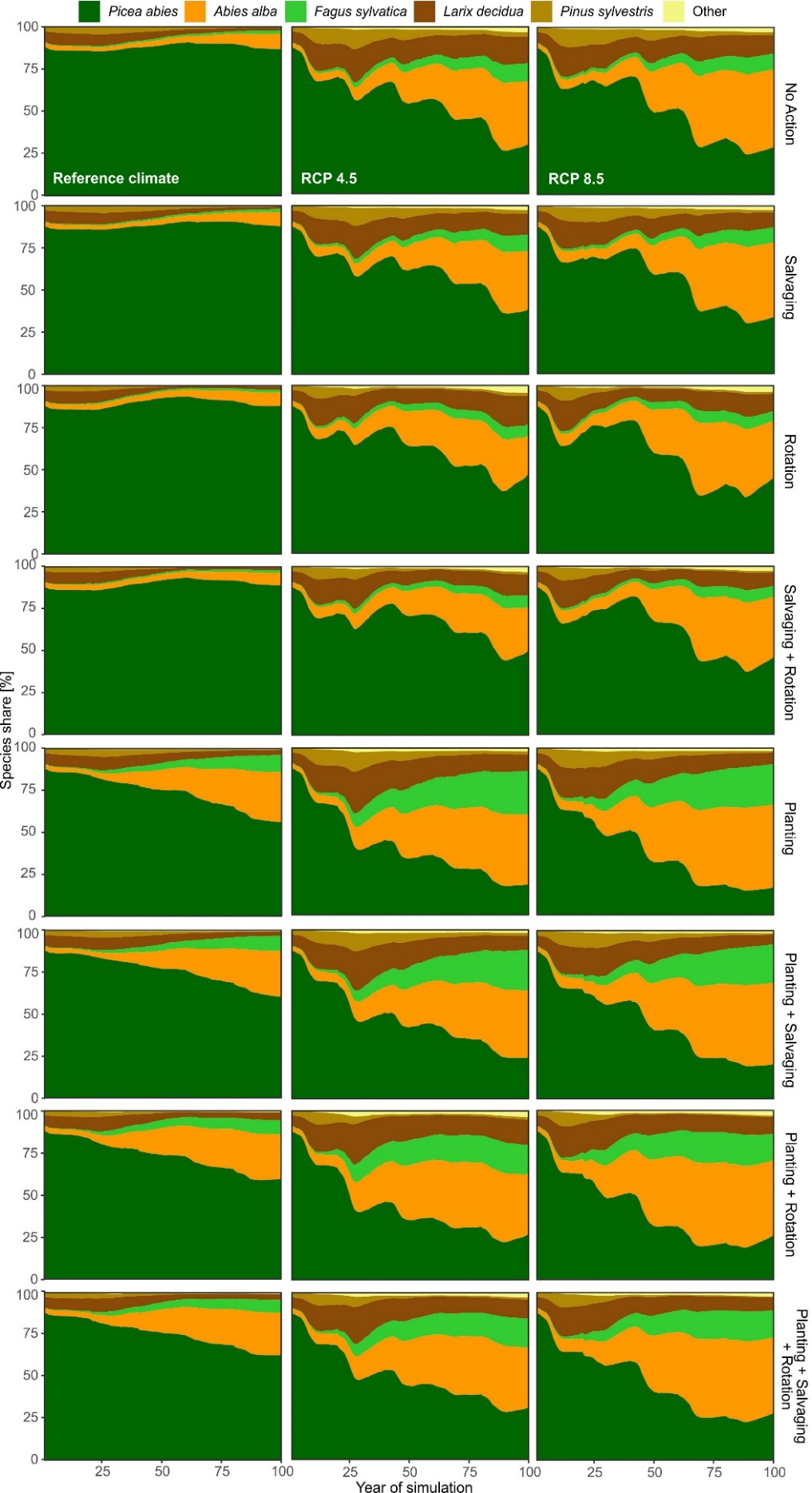
**Supplement F: Tree species composition in the study landscape simulated under different climates and management regimes**

Fig. F1 Tree species composition in the study landscape simulated under different climates and management regimes. Managements description is in Table 1 in the text.
